# An umbrella review of systematic reviews and meta-analyses of observational investigations of obstructive sleep apnea and health outcomes

**DOI:** 10.1007/s11325-021-02384-2

**Published:** 2021-04-24

**Authors:** Weiwei Chen, Yuting Li, Liliangzi Guo, Chenxing Zhang, Shaohui Tang

**Affiliations:** Department of Gastroenterology, The First Affiliated Hospital, Jinan University, Guangzhou, People’s Republic of China

**Keywords:** Obstructive sleep apnea, Health, Umbrella review, Meta-analysis

## Abstract

**Purpose:**

The previous analysis of systematic reviews and meta-analyses have illustrated that obstructive sleep apnea (OSA) is correlated with multiple health outcomes. In the present research, our main aim was to execute an umbrella review to assess the available evidence for the associations between OSA and health outcomes.

**Methods:**

Herein, a meta-analysis of previous observational investigations that have reported associations between OSA and health outcomes in all human populations and settings was performed. We used these studies to execute an umbrella review of available meta-analyses and systematic reviews.

**Results:**

Sixty-six articles comprising 136 unique outcomes were enrolled in this analysis. Of the 136 unique outcomes, 111 unique outcomes had significant associations (*p* < 0.05). Only 7 outcomes (coronary revascularization after PCI, postoperative respiratory failure, steatosis, alaninetrans aminase (ALT) elevation, metabolic syndrome (MS), psoriasis, and Parkinson’s disease) had a high quality of evidence. Twenty-four outcomes had a moderate quality of evidence, and the remaining 80 outcomes had a weak quality of evidence. Sixty-nine outcomes exhibited significant heterogeneity. Twenty-five outcomes exhibited publication bias. Sixty-three (95%) studies showed critically low methodological quality.

**Conclusion:**

Among the 66 meta-analyses exploring 136 unique outcomes, only 7 statistically significant outcomes were rated as high quality of evidence. OSA may correlate with an increased risk of coronary revascularization after PCI, postoperative respiratory failure, steatosis, ALT elevation, MS, psoriasis, and Parkinson’s disease.

## Introduction

Obstructive sleep apnea (OSA) is a prevalent but treatable chronic sleep disorder that is determined through episodes of sleep apnea and hypopnea during sleep and results in recurrent episodes of hypercapnia and hypoxemia [1–3]. OSA has a prevalence of between 5 and 20% depending on the population surveyed and the definition utilized [4, 5]. The prevalence is also increasing due to an increase in body mass index which is one of its major predisposing factors. Apart from causing uncomfortable symptoms such as headache [6] and attention deficit [7], earlier studies indicated that OSA also contributed to the advancement of several diseases including hypertension [8], cardiovascular disease [9, 10], and diabetes [11]. Recent studies have drawn consistent conclusions [12–14]. Recently, a great number of researches have explored the correlation between OSA and other diseases. Multiple investigations and meta-analyses have illustrated that OSA poses a threat to human health because it increases the risk of various diseases, including cancers [15–17], depression [18], laryngopharyngeal reflux disease [19], metabolic disease [20], Parkinson’s disease [21], and chronickidney disease (CKD) [22].

These studies suggest a possible causal relationship between OSA and different health outcomes, indicating that OSA has a bad influence on human health. However, several factors are known to decrease the validity and strength of reported evidence including publication bias, protocol design flaws, or inconsistencies of studies. Currently, there have been no systematic reviews that have accurately summarized and critically appraised existing studies. In the current study, an umbrella review was executed to comprehensively evaluate published systematic reviews and meta-analyses of observational researches that reported associations between OSA and health information. This work can provide important guidance in the diagnosis and treatment of OSA.

## Materials and methods

The protocol of the research was registered with PROSPERO (registration number: CRD42020220015) before the umbrella review began. A systematic exploration of the literature search was accomplished in compliance with the Preferred Reporting Items for Systematic Reviews and Meta-Analyses (PRISMA) protocols [23].

### Literature search

From initiation until November 23, 2020, literature searches were performed using online databases such as Embase, PubMed, the Cochrane Database of Systematic Reviews, and the Web of Science. Literature searches were independently conducted by two researchers (CZ and LG). The search terms applied were (“obstructive sleep apnea” OR “obstructive sleep apnea–hypopnea” OR “OSA” OR “OSAH”) AND (Meta-Analysis[ptyp] OR metaanaly*[tiab] OR meta-analy*[tiab] OR Systematic review [ptyp] OR “systematic review”[tiab]). The references were manually screened to identify eligible articles to be included in the study. The article titles, abstracts, and the complete manuscripts of the identified paper were then further assessed. A discussion was used to resolve potential discrepancies; ST acted as an arbiter to deal with discrepancies that could not be resolved by discussion among the investigators.

### Eligibility criteria and exclusion criteria

The eligibility of articles was based on a systematic search by the authors to identify the most pertinent studies. Only systematic reviews or meta-analyses on the basis of the epidemiological studies performed in humans were considered in the analysis. Diagnostic trials and meta-analyses of interventional trials were not performed as part of the current study. Furthermore, the abstracts of the conference on review questions were not included in the final analysis. The final systematic reviews and meta-analyses that were analyzed had to include the data of pooled summary effects(i.e., relative risks (RRs); odds ratios (ORs); hazard ratios (HRs); mean difference (MD); weighted mean difference (WMD); standard mean difference (SMD); and their 95% confidence intervals (CIs)), number of included researches, number of participants and cases, heterogeneity, and publication bias. Whenever more than one meta-analysis was executed using on the basis of the same outcome, the agreement with the main conclusions reported in the study were verified. When the reported conclusions were conflicting, the meta-analysis with the greatest number of investigations was considered.

### Data extraction

For investigations to be eligible for inclusion in the meta-analysis, two researchers (WC and YL) independently extracted data from the articles. This included the first author, the number of included investigations, the year of publication, the study design, the whole numbers of cases, and participants. The reported relative summary risk evaluates (ORs, RRs, HRs, SMD, WMD, or MD) and the corresponding 95% CIs were extracted, for each eligible systematic review and meta-analysis. The values of *p* for the total pooled effects, Cochran *Q* measurement, Egger’s measurement, and *I*^2^ were extracted. Discrepancies in the analyses were resolved by discussion among the investigators.

### Assessment of methodological quality

Two investigators (WC and YL) independently assessed the quality of the methods reported in the studies. This was performed using a 16-criteria checklist included in AMSTAR 2 [24]. AMSTAR 2 is a fundamental revision of the original instrument of AMSTAR which was devised to evaluate systematic reviews that included randomized controlled experiments. The AMSTAR 2 score is categorized as high in studies that have no or one noncritical weakness, moderate in surveys with more than one noncritical weakness, low when the study has only one serious flaw without or with noncritical weaknesses, and seriously low when a study has more than one serious flaw without or with nonserious weaknesses. Discrepancies between the AMSTARS 2 scores for the articles were resolved by discussion between the investigators.

### Assessment of the evidence quality

Two investigators (WC and YL) independently evaluated the quality of the evidence conforming to the parameters that have previously been applied in various fields [25–28]. Discrepancies were resolved by discussion. First, *p* value for the estimate < 0.001 [29, 30] and more than 1000 cases of the disease, which indicated fewer false-positive results. Second, *I*^2^ < 50% and *p* value for Cochran *Q* test > 0.10, which indicated consistency of results. Third, *p* value for Egger’s test > 0.10, which exhibited no evidence of small-study impacts. When all of the above criteria were satisfied, the strength of the epidemiologic evidence was rated as high. When 1 of the criterion was not satisfied and the *p* value for the estimate was < 0.001, the strength of the epidemiologic evidence was rated as moderate. Then, the rest was defined as weak (*p* < 0.05). The value of *p* for the evaluation can be assessed from the 95% confidence interval of the pooled impact estimate utilizing an established method [31] if it was not directly reported in the article.

### Data analysis

From each of the published studies, the outcome data of the available meta-analyses was extracted along with the estimated summary effect at the corresponding 95% CI. The total impacts of the pooled meta-analysis were considered significant when the *p*-value was < 0.05. Heterogeneity was appraised by the *I*^2^ test and *Q* test, publication bias was estimated by utilizing Egger’s test, and both were considered significant at *p* < 0.1. Studies that did not have the heterogeneity or publication bias results were reanalyzed if raw data were available.

## Results

### Characteristics of the meta-analyses

**Fig. 1:**
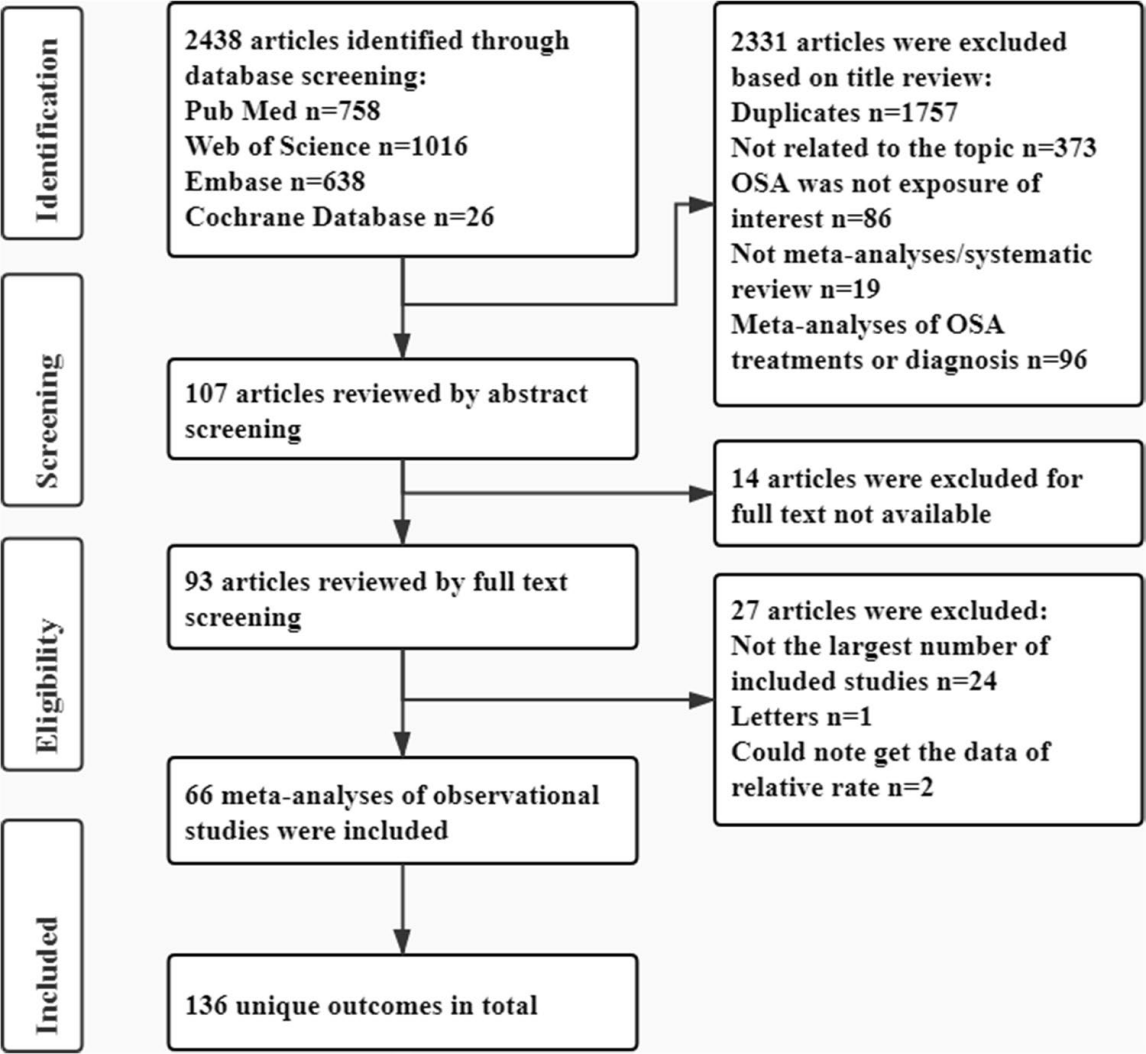
Flowchart of the selection procedure

**Fig. 2:**
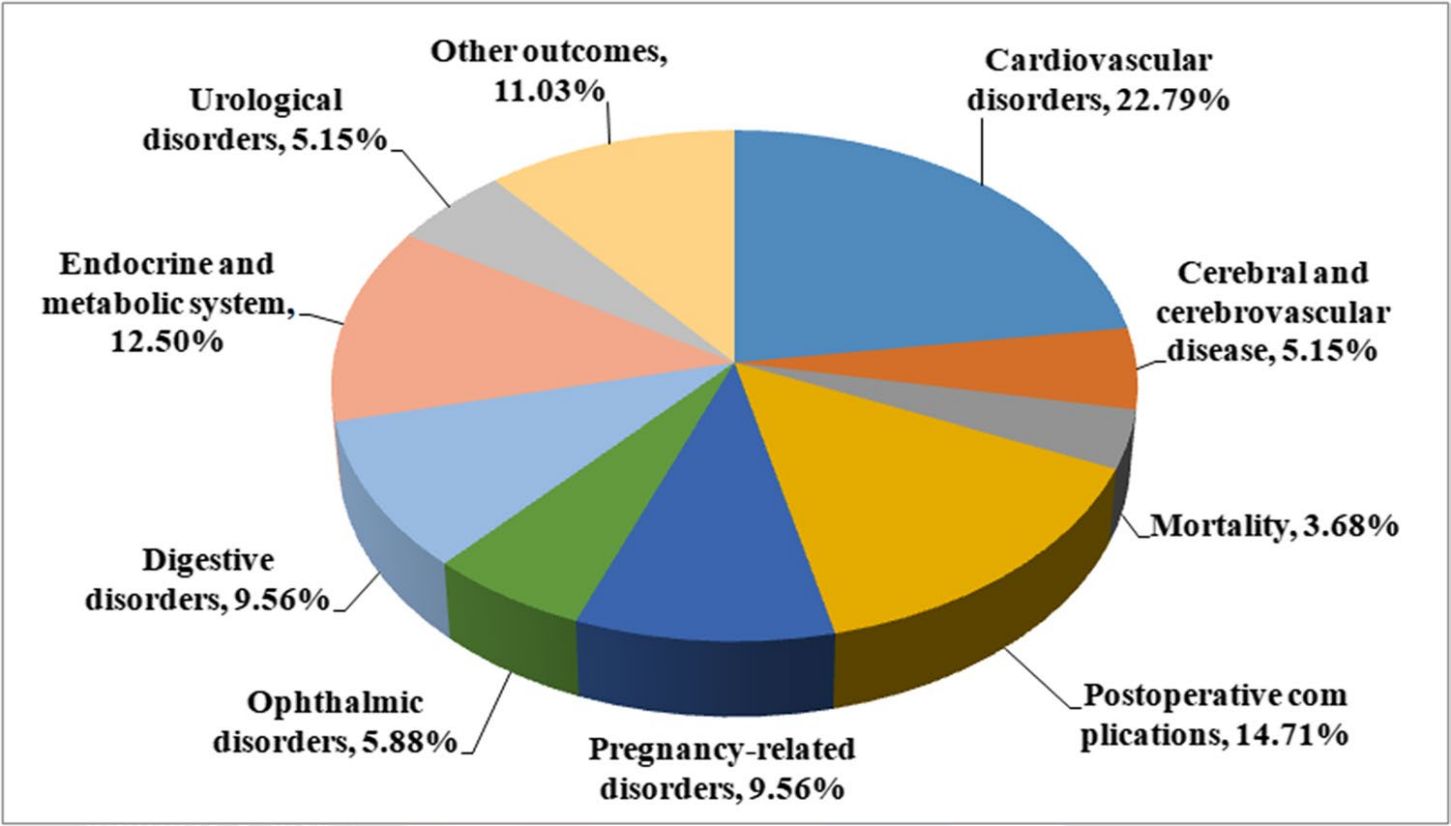
Map of achievements related to OSA

The outcomes of the systematic investigation and the selection of eligible investigations are summarized in Fig. 1. Overall, 1972 articles were searched from which 66 meta-analyses of observational investigations were identified that had 136 unique outcomes [21, 22, 32–95]. The 66 eligible non-overlapping meta-analyses had publication dates ranging from 2009 to 2020 and are summarized in Table 1. The median number of primary investigations per evidence synthesis was 7 (range 2–64). Furthermore, 1 meta-analysis [54] lacked the data of both participants and cases, and 2 meta-analyses [52, 95] lacked the data of cases. Among the meta-analyses identified in this study, the median number of cases was 900 (88–3,117,496) and the median number of participants was 2962 (170–56,746,100). An extensive range of data were reported such as cardiovascular disorders (*n* = 31), cerebral and cerebrovascular disease (*n* = 7), mortality (*n* = 5), postoperative complications (*n* = 20), pregnancy-related disorders (*n* = 13), ophthalmic disorders (*n* = 8), digestive disorders (*n* = 13), endocrine and metabolic system disorders(*n* = 17), urological disorders (*n* = 7), and other data (*n* = 15) (Fig. 2).

**Table 1:** Associations between OSA and multiple heath outcomes

Outcomes	Publication	Number of studies		Number of participants		Number of cases			Type of metric			Relative risk (95% CI)			*P* value*			*P* value ^#^				*I*^2^ (%)		*P* value^※^	Whether exist publication bias		
Cardiovascular disorders																											
Aortic dissection	Xiushi Zhou (2018)	1 cohort study, 2 case–control studies		55,911		16,019			OR			1.60 (1.01–2.53)				0.04		0.44				0		0.58	No		
Cardiovascular disease(CVD)	Xia Wang (2013)	11 cohort studies		25,594		2628			RR			1.79 (1.47–2.18)				< 0.001		0.131				31.5		0.028	Yes		
Stroke	Min Li (2014)	10 cohort studies		18,609		678			RR			2.10 (1.50–2.93)				< 0.001		0.04				47.5		0.288^&^	No		
Ischemic heart disease(IHD)	Wuxiang Xie (2014)	6 cohort studies		1083		625			RR			1.83 (1.15–2.93)				0.011		0.111				44.2		0.006	Yes		
Coronary heart disease(CHD)	Chengjuan Xie (2017)	6 cohort studies		18,022		15,562			RR			1.63 (1.18–2.26)				0.003		0.061^&^				52.7^&^		0.145^&^	No		
Major adverse cardiac events (MACEs)	Chengjuan Xie (2017)	9 cohort studies		18,022		15,562			RR			2.04 (1.56–2.66)				< 0.001		0.021				55.7		0.132	No		
Atrial fibrillation	Irini Youssef (2018)	4 cross-sectional studies, 5 cohort studies		19,837		12,255			OR			2.12 (1.84–2.43)				< 0.001		0.004				64.42		0.097^&^	Yes		
Resistant hypertension	Haifeng Hou (2018)	6 case -control studies		1465		925			OR			2.84 (1.70–3.98)				< 0.001		0.816				0		0.187^&^	No		
Essential hypertension	Haifeng Hou (2018)	2 case–control studies, 5 cohort studies		7102		4513			OR			1.80 (1.54–2.06)				< 0.001		0.221				26		0.0526^&^	Yes		
Atrial fibrillation recurrence after catheter ablation	Chee Yuan Ng (2011)	6 observational studies		3995		958			RR			1.25 (1.08–1.45)				0.003		0.008				49		0.879^&^	No		
major adverse cardiovascular event (MACE) after PCI	Xiao Wang (2018)	9 observational studies		2755		1581			RR			1.96 (1.36–2.81)				< 0.001		0.02				54		0.002	Yes		
Stroke after PCI	Xiao Wang (2018)	6 observational studies		2110		1254			RR			1.55 (0.90–2.67)				0.11		0.62				0		0.149^&^	No		
Myocardial infarction (MI) after PCI	Hua Qu (2018)	6 observational studies		2342		1112			OR			1.59 (1.14–2.23)				0.007		0.32				15		0.655^&^	No		
Coronary revascularization after PCI	Hua Qu (2018)	7 observational studies		2415		1163			OR			1.57 (1.23–2.01)				< 0.001		0.7				0		0.483^&^	No		
Re-admission for heart failure after PCI	Hua Qu (2018)	4 observational studies		1774		793			OR			1.71 (0.99–2.96)				0.06		0.86				0		0.254^&^	No		
Left ventricular hypertrophy (LVH)	Cesare Cuspidi (2020)	9 observational studies		3244		1802			OR			1.70 (1.44–2.00)				< 0.001		< 0.001				60		0.0876^&^	Yes		
Left ventricular diastolic diameter (LVEDD)	LeiYu (2019)	13 observational studies		882		563			WMD			1.24 (0.68, 1.80)				< 0.001		0.658				0		0.431	No		
Left ventricular systolic diameter (LVESD)	LeiYu (2019)	11 observational studies		630		396			WMD			1.14 (0.47, 1.81)				0.001		0.696				0		0.722	No		
Left ventricular mass(LVM)	LeiYu (2019)	6 observational studies		432		304			WMD		35.34 (20.67, 50.00)					< 0.001		< 0.001				79.1		0.914	No		
Leftventricular ejection fraction (LVEF)	LeiYu (2019)	15 observational studies		1104		710			WMD		− 3.01 (− 1.90, − 0.79)					0.001		< 0.001				64.7		0.048	Yes		
Left atrial diameter (LAD)	LeiYu (2019)	7 observational studies		468		311			WMD		2.13 (1.48, 2.77)					< 0.001		0.408				2.2		0.072	Yes		
Left atrial diameter volume index (LAVI)	LeiYu (2019)	3 observational studies		228		159			WMD		3.96 (3.32, 4.61)					< 0.001		0.445				0		0.735	No		
Right ventricular internal diameter (RVID)	Abdirashit Maripov (2017)	16 observational studies		1498		902			WMD		2.49 (1.62, 3.37)					< 0.001		< 0.001				96.8		0.001	Yes		
Right ventricular free wall thickness (RVWT)	Abdirashit Maripov (2017)	9 observational studies		976		579			WMD		0.82 (0.51, 1.13)					< 0.001		< 0.001				95.6		0.671	No		
Right ventricular myocardial performance index(RV MPI)	Abdirashit Maripov (2017)	14 observational studies		1298		864			WMD		0.08 (0.06, 0.10)					< 0.001		< 0.001				84.1		0.15	No		
Tricuspid annular systolic velocity (RV S′)	Abdirashit Maripov (2017)	14 observational studies		1030		639			WMD		− 0.95 (− 0.32, − 1.59)					0.003		< 0.001				88.4		0.347	No		
Tricuspid annular plane systolic excursion (TAPSE)	Abdirashit Maripov (2017)	11 observational studies		1033		655			WMD		− 1.76 (− 0.78, − 2.73)					< 0.001		< 0.001				89.3		0.462	No		
Right ventricular fractional area change (RA FAC)	Abdirashit Maripov (2017)	6 observational studies		661		422			WMD		− 3.16 (− 0.73, − 5.60)					0.011		< 0.001				80.2		0.006	Yes		
Epicardial adipose tissue (EAT) thickness	Guang Song (2020)	9 observational studies		1178		898			WMD		0.95 (0.73, 1.16)					< 0.001		< 0.001				64.7		0.549	No		
Coronary flow reserve (CFR)	Rui-Heng Zhang (2020)	1 case–control study, 4 cross-sectional studies		1336		829			WMD		’ − 0.78 (− 0.32, − 1.25)					< 0.001		< 0.001				84.4		0.49	No		
Systolic blood pressure (SBP)	De-Lei Kong (2016)	2 cross-sectional studies, 3 cohort studies, 1 case–control studies		1046		534			SMD		0.56 (0.40, 0.71)					< 0.001		0.132				41.03		NA	NA		
Cerebral and cerebrovascular disease																											
Cerebral white matter changes	Bo-Lin Ho (2018)	10 observational studies		1582		818			OR		2.06 (1.52–2.80)					< 0.001		0.025				48.5		0.338		No	
Cerebrovascular (CV) disease	Zesheng Wu (2018)	15 cohort studies		3,120,368		3,117,496			HR		1.94 (1.31–2.89)					0.001		< 0.001				90.3		> 0.05		No	
White matter hyperintensities (WMH)	Yuhong Huang (2019)	11 cross-sectional studies, 2 case–control studies		4412		2065			OR		2.23 (1.53–3.25)					< 0.001		< 0.001				80.3		< 0.01		Yes	
Silent brain infarction (SBI)	Yuhong Huang (2019)	9 cross-sectional studies, 2 case–control studies, 1 cohort study		3353		1893			OR		1.54 (1.06–2.23)					0.023		0.018				52		0.605		No	
Cerebral microbleeds (CMBs)	Yuhong Huang (2019)	3 cross-sectional studies		342		271			OR		2.17 (0.61–7.73)				0.234			< 0.01				60.2		NA		Unclear	
Perivascular spaces (PVS)	Yuhong Huang (2019)	2 cross-sectional studies		267		152			OR		1.56 (0.28–8.57)				0.623			< 0.01				69.5		NA		NA	
Asymptomatic lacunar infarction (ALI)	AnthipaChokesuwattanaskul (2019)	6 cross-sectional studies, 1 cohort study		1756		713			OR		1.78 (1.06–3.01)				0.03			0.128^&^				41		0.43		No	
Mortality																											
All-cause mortality	Lei Pan (2016)	12 cohort studies		34,382		18,139			HR		1.26 (1.09–1.43)				0.001		< 0.001			70.4				0.003		Yes	
Cardiovascular mortality	Xiahui Ge (2013)	4 cohort studies		5228		239			RR		2.21 (1.61–3.04)				< 0.001		0.418			0				0.448		No	
All-cause death after PCI	Xiao Wang (2018)	4 cohort studies		1919		1154			RR		1.70 (1.05–2.77)				0.03		0.71			0				0.176^&^		No	
Cardiac death after PCI	Hua Qu (2018)	7 cohort studies		2465		1187			OR		2.05 (1.15–3.65)				0.01		0.96			0				0.828^&^		No	
Cancer mortality	Xiaobin Zhang (2017)	3 cohort studies		7346		179			HR		1.38 (0.79–2.41)				0.257		0.004			66.1				0.205		No	
Postoperative complications																											
Postoperative respiratory failure	Faizi Hai BA (2013)	12 cohort studies		5611		2390			OR		2.42 (1.53–3.84)				< 0.001		0.39		5					0.28	No		
Postoperative cardiac events	Faizi Hai BA (2013)	11 cohort studies		3781		2109			OR		1.63 (1.16–2.29)				0.005		0.7		0					0.187^&^	No		
Postoperative desaturation	R. Kaw (2012)	11 cohort studies		3645		1764			OR		2.27 (1.20–4.26)				0.01		< 0.001		68					0.04^&^	Yes		
Postoperative ICU transfer	R. Kaw (2012)	9 cohort studies		5743		2062			OR		2.81 (1.46–5.43)				0.002		0.02		57					0.033^&^	Yes		
Postoperative composite endpoints of postoperative cardiac or cerebrovascular complications	Ka Ting Ng (2020)	12 observational studies		2,003,694		126,027			OR		1.44 (1.17–1.78)				< 0.001		NA		89					NA	Unclear		
Postoperative myocardial infarction	Ka Ting Ng (2020)	8 observational studies		714,650		NA			OR		1.37 (1.19–1.59)				< 0.001		NA		36					NA	Unclear		
Postoperative congestive cardiac failure	Ka Ting Ng (2020)	3 observational studies		2104		NA			OR		3.16 (1.02–9.81)				0.05		NA		0					NA	Unclear		
Postoperative atrial fibrillation	Ka Ting Ng (2020)	6 observational studies		1,463,449		NA			OR		1.50 (1.30–1.73)				< 0.001		NA		87					NA	Unclear		
Postoperative cerebrovascular accident	Ka Ting Ng (2020)	5 observational studies		1,641,495		NA			OR		1.09 (0.75–1.60)				0.65		NA		61					NA	Unclear		
Postoperative composite endpoints of pulmonary complications	Ka Ting Ng (2020)	8 observational studies		1,983,748		NA			OR		2.52 (1.92–3.31)				< 0.001		NA		96					NA	Unclear		
Postoperative pneumonia	Ka Ting Ng (2020)	10 observational studies		2,675,205		NA			OR		1.66 (1.17–2.35)				0.004			NA	96					NA	Unclear		
Postoperative reintubation	Ka Ting Ng (2020)	9 observational studies		2,061,268		NA			OR		2.29 (0.90–5.82)				0.08			NA	99					NA	Unclear		
Postoperative in-hospital mortality	Ka Ting Ng (2020)	6 observational studies		2,497,794		NA			OR		0.86 (0.42–1.76)				0.68			NA	94					NA	Unclear		
Postoperative 30-day mortality	Ka Ting Ng (2020)	6 observational studies		616,754		NA			OR		1.27 (1.03–1.57)				0.02			NA	0					NA	Unclear		
Postoperative acute kidney injury	Ka Ting Ng (2020)	5 observational studies		1,724,932		NA			OR		2.41 (1.93–3.02)				< 0.001			NA	92					NA	Unclear		
Postoperative delirium	Ka Ting Ng (2020)	6 observational studies		2346		NA			OR		2.45 (1.50–4.01)				< 0.001			NA	2					NA	Unclear		
Postoperative venoembolism	Ka Ting Ng (2020)	10 observational studies		2,100,013		NA			OR		1.63 (1.17–2.27)				0.004			NA	94					NA	Unclear		
Postoperative surgical site infection	Ka Ting Ng (2020)	5 observational studies		2962		NA			OR		1.30 (0.93–1.83)				0.13			NA	0					NA	Unclear		
Postoperative bleeding	Ka Ting Ng (2020)	3 observational studies		18,712		NA			OR		1.10 (0.40–3.01)				0.85			NA	63					NA	Unclear		
Postoperative length of hospital stay	Ka Ting Ng (2020)	15 observational studies		1,569,278		NA			MD		0.09 (0.00–0.17)				0.04			NA	96					NA	Unclear		
Pregnancy-related disorders																											
Gestational diabetes mellitus (GDM)	Xinge Zhang (2020	6 cohort studies		2,522,547		139,559			RR		1.60 (1.21–2.12)				0.004			0.003	69.2					0.4829	No		
C-section	Lina Liu (2019)	6 observational studies		NA		NA			OR		1.42 (1.12–1.79)				< 0.001			< 0.001	86.5					NA	Unclear		
Pregnancy-related prolonged hospital stay	Lina Liu (2019)	3 observational studies		NA		NA			OR		1.94 (0.88–4.28)				0.1			< 0.001	98.6					NA	Unclear		
Pregnancy-related wound complication	Lina Liu (2019)	3 observational studies		NA		NA			OR		1.87 (1.56–2.24)				< 0.001			0.883	0					NA	Unclear		
Pregnancy-related pulmonary edema	Lina Liu (2019)	3 observational studies		NA		NA			OR		6.35 (4.25–9.50)				< 0.001			0.294	18.2					NA	Unclear		
Small for gestational age	Lina Liu (2019)	4 observational studies		NA		NA			OR		1.26 (0.80–2.01)				0.321			0.01	73.8					NA	Unclear		
Stillbirth	Lina Liu (2019)	3 observational studies		NA		NA			OR		1.12 (0.85–1.49)				0.413			0.572	0					NA	Unclear		
Poor fetal growth	Lina Liu (2019)	4 observational studies		NA		NA			OR		1.15 (0.98–1.34)				0.091			0.266	24.3					NA	Unclear		
Gestational hypertension	Liwen Li (2018)	4 cross-sectional studies, 7 cohort studies		56,731,077		19,047			OR		1.80 (1.28–2.52)				0.001			0.72	0					0.649^&^	No		
Preeclampsia	Liwen Li (2018)	2 cross-sectional studies, 7 cohort studies		56,097,993		19,776			OR		2.63 (1.87–3.70)				< 0.001			< 0.01	78					0.797^&^	No		
Preterm birth	Liwen Li (2018)	2 cross-sectional studies, 3 cohort studies		56,746,100		18,337			OR		1.75 (1.21–2.55)				0.003			< 0.01	90					0.931^&^	No		
Birth weight	Liwen Li (2018)	4 cohort studies		4311		1387			WMD		− 47.46 (− 242.09, 147.16)				0.281			< 0.01	93					NA	No^$^		
Neonatal intensive care unit (NICU) admission	Ting Xu (2014)	4 cohort studies		757		177			RR		2.65 (1.86–3.76)				< 0.001			0.235	29.6					0.063^&^	Yes		
Ophthalmic disorders																											
Diabetic retinopathy (DR)	Zhenliu Zhu (2017)		6 case -control studies	1092		608			OR			2.01 (1.49–2.72)				< 0.001		0.062			52.4		0.112^&^				No
Keratoconus	Marco Pellegrini (2020)		4 case–control studies, 1 cohort study	33,844		16,922			OR			1.84 (1.16–2.91)				0.009		0.003			74.6		0.07				Yes
Glaucoma	Xinhua Wu (2015)		12 observational studies	36,909		11,765			OR			1.65 (1.44–1.88)				< 0.001		0.06			43		0.335				No
Floppy eyelid syndrome (FES)	Leh-Kiong Huon (2016)		7 cross-sectional studies	902		337			OR			4.70 (2.98–7.41)				< 0.001		0.129^&^			39.3^&^		0.379^&^				No
Nonarteritic anterior ischemic optic neuropathy (NAION)	Yong Wu (2015)		4 cohort studies, 1 case–control study	5916		164			OR			6.18 (2.00–19.11)				0.002		0.002			77		0.35				No
Central serous chorioretinopathy (CSCR)	Chris Y.Wu (2018)		6 case–control studies	7238		1479			OR			1.56 (1.16–2.10)				0.003		0.237			26.3		0.281				No
retinal nerve fiber layer (RNFL) thickness	Cheng-Lin Sun (2016)		8 case–control studies	1237		763			WMD			− 2.92 (− 4.61, − 1.24)				0.001		0.017			59.1		0.929				No
Choroidal thickness	Chris Y.Wu (2018)		9 case–control studies	778		514			WMD			25.52 (− 78.79, − 27.76)				0.824		0.001			98.6		0.137				No
Digestive disorders																											
Gastroesophageal reflux disease	Zeng-Hong Wu (2019)		1 case–control study, 6 cross-sectional studies	2699		1452			OR			1.75 (1.18–2.59)				0.006		0.04	54				0.052				Yes
Steatosis	Shanshan Jin (2018)		3 cohort studies, 1 cross-sectional study	1635		1375			OR			3.19 (2.34–4.34)				< 0.001		0.677	0				0.89				No
Lobular inflammation	Shanshan Jin (2018)		3 cohort studies	350		205			OR			2.85 (1.8–-4.49)				< 0.001		0.994	0				0.469				No
Ballooning degeneration	Shanshan Jin (2018)		3 cohort studies	350		205			OR			2.29 (1.36–3.84)				0.002		0.774	0				0.888				No
NAFLD activity score(NAS)	Shanshan Jin (2018)		3 cohort studies	350		205			OR			1.63 (0.68–3.86)				0.271		0.259	25.9				0.839				No
NAFLD defined by liver histology	G. Musso (2013)		8 cross-sectional studies	994		537			OR			2.01 (1.36–2.97)				< 0.001		0.4	4				0.303^&^				No
NAFLD defined by radiology	G. Musso (2013)		6 cross-sectional studies	561		269			OR			2.99 (1.79–4.99)				< 0.001		0.33	13				0.433^&^				No
NAFLD defined by AST elevation	G. Musso (2013)		11 cross-sectional studies	746		368			OR			2.36 (1.46–3.82)				< 0.001		0.99	0				0.65^&^				No
NAFLD defined by ALT elevation	G. Musso (2013)		14 cross-sectional studies	1833		938			OR			2.60 (1.88–3.61)				< 0.001		0.74	0				0.179^&^				No
Nonalcoholic steatohepatitis(NASH)	G. Musso (2013)		10 cross-sectional studies	1114		589			OR			2.37 (1.59–3.51)				< 0.001		0.81	0				0.404^&^				No
Fibrosis	G. Musso (2013)		10 cross-sectional studies	1114		589			OR			2.16 (1.45–3.20)				< 0.001		0.67	0				0.778^&^				No
Alanine transaminase (ALT)	Shanshan Jin (2018)		7 cohort studies, 1 cross-sectional study	2059		1684			SMD			0.21 (0.11, 0.31)				< 0.001		0.672	0				0.468				No
Aspartate transaminase (AST)	Shanshan Jin (2018)		7 cohort studies, 1 cross-sectional study	2059		1684			SMD			0.07 (− 0.03, 0.17)				0.152		0.918	0				< 0.05				Yes
Endocrine and metabolic system disorders																											
Type 2 diabetes (T2DM)	Ranran Qie (2020)		16 cohort studies	338,912		19,355			RR			1.40 (1.32–1.48)			< 0.001			0.045	40.8				0.221^&^				No
Metabolic syndrome (MS)	Shaoyong Xu (2015)		15 cross-sectional studies	4161		2457			OR			2.87 (2.41–3.42)			< 0.001			0.23	20				0.232				No
Fasting blood glucose (FBG)	De-Lei Kong (2016)		3 cross-sectional studies, 5 cohort studies, 2 case–control studies	2053		1296			SMD			0.35 (0.18, 0.53)			< 0.001			0.008	59.69				NA				No^$^
Total cholesterol (TC)	Rashid Nadeem (2014)		63 observational studies	18,111		NA			SMD			0.267 (0.146, 0.389)			0.001			NA	NA				NA				No^$^
Low-density lipoprotein (LDL)	Rashid Nadeem (2014)		50 observational studies	13,894		NA			SMD			0.296 (0.156, 0.436)			0.001			NA	NA				NA				No^$^
High-density lipoprotein (HDL)	Rashid Nadeem (2014)		64 observational studies	18,116		NA			SMD			− 0.433 (− 0.604, − 0.262)			< 0.001			NA	NA				NA				No^$^
Triglyceride (TG)	Rashid Nadeem (2014)		62 observational studies	17,831		NA			SMD			0.603 (0.431, 0.775)			< 0.001			NA	NA				NA				No^$^
Adiponectin	Mi Lu (2019)		20 case–control studies	1356		878			SMD			′ − 0.71 (− 0.92, − 0.49)			< 0.001			< 0.01	73				0.09				Yes
Oxidized low-density lipoprotein (Ox-LDL)	Reza Fadaei (2020)		8 case -control studies	623		391			SMD			0.95 (0.24, 1.67)			0.009			< 0.001	94.1				< 0.161				No
Fibrinogen	Fang Lu (2019)		25 observational studies	3792		1480			WMD			0.38 (0.29, 0.47)			< 0.001			< 0.001	80.3				0.208				No
Homocysteine	Kun Li (2017)		10 observational studies	773		457			MD			2.40 (0.60, 4.20)			0.009			< 0.001	96				0.947				No
Advanced glycation end products (AGEs)	Xingyu Wu (2018)		5 cross-sectional studies	670		323			SMD			0.98 (0.69, 1.27)			< 0.001			0.08	51				NA				No^$^
Plasma renin activity(PRA)	Ze-Ning Jin (2016)		5 case–control studies	300		180			MD			0.17 (− 0.22, 0.55)			0.4			< 0.001	82				NA				Unclear
Plasma renin concentration(PRC)	Ze-Ning Jin (2016)		5 case–control studies	170		101			MD			0.95 (− 0.58, 2.48)			0.23			0.001	78				NA				Unclear
Angiotensin II(AngII)	Ze-Ning Jin (2016)		7 case–control studies	384		207			MD			3.39 (2.00, 4.79)			< 0.001			< 0.001	95				0.167				No
Aldosterone	Ze-Ning Jin (2016)		9 case–control studies	474		265			MD			0.95 (− 0.16, 2.07)			0.09			< 0.001	78				0.622				No
Serum vitamin D	Xiaoyan Li (2020)		6 case–control studies, 21 cross-sectional studies, 2 cohort studies	6298		4209			SMD			′ − 0.84(− 1.14, − 0.54)			< 0.001			< 0.001	95				NA				No^$^
Urological disorders																											
Diabetic kidney disease (DKD)	Wen Bun Leong (2016)		7 cross-sectional studies		1877		1159			OR			1.59 (1.16–2.18)		0.004			0.224^&^	26.8				0.684^&^				No
Microalbuminuria	Tongtong Liu (2020)		4 cross-sectional studies		667		415			RR			2.32 (1.48–3.62)		< 0.001			0.578	0				0.55				No
Chronic kidney disease (CKD)	Der-Wei Hwu (2017)		2 cohort studies, 16 cross-sectional studies		7090		3720			OR			1.77 (1.37–2.29)		< 0.001			< 0.001^&^	87.2^&^				0.011^&^				Yes
Serum uric acid level	Tingting Shi (2019)		14 observational studies		5219		2656			WMD			50.25 (36.16,64.33)		< 0.001			< 0.001	91.2				0.001				Yes
Serum cystatin C	Tongtong Liu (2020)		7 cross-sectional studies		1412		274			SMD			0.53 (0.42,0.64)		< 0.001			0.16	33.7				0.111				No
Estimated glomerular filtration rate (eGFR)	Tongtong Liu (2020)		13 cross-sectional studies		3344		657			SMD			− 0.19 (− 0.27, − 0.12)		0.001			0.057	33.1				0.516				No
Albumin/creatinine ratio(ACR)	Tongtong Liu (2020)		3 cross-sectional studies		740		88			WMD			0.71 (0.58, 0.84)		< 0.001			0.003	69.2				0.574				No
Other outcomes																											
Diabetic neuropathy	Xiandong Gu (2018)		11 case -control studies		1842			840		OR				1.84 (1.18–2.87)	0.007			< 0.01	68.6				0.13				No
Psoriasis	Tzong-Yun Ger (2020)		3 cohort studies		5,544,674			42,656		RR				2.52 (1.89–3.36)	< 0.001			0.95	0				0.545				No
Nocturia	Jiatong Zhou (2019)		3 cohort studies, 8 case–control studies, 2 cross-sectional studies		9924			406		RR				1.41 (1.26–1.59)	< 0.001			0.001	63.3				0.076				Yes
Allergic rhinitis	Yuan Cao (2018)		1 cross-sectional study, 2 case–control studies, 1 cohort study		1283			371		OR				1.73 (0.94–3.20)	0.078			0.023	64.8				0.977				No
Parkinson’s disease	A-Ping Sun (2020)		4 cohort studies, 1 case–control study		83,449			26,070		HR				1.59 (1.36–1.85)	< 0.001			0.17	40				0.186				No
Erectile dysfunction	Luhao Liu (2015)		1 cohort study, 3 case–control studies, 1 cross-sectional study		834			532		RR				1.82 (1.12–2.97)	0.016			0.002	76.5				0.077				Yes
Female sexual dysfunction	Luhao Liu (2015)		2 case–control studies, 2 cohort studies		438			149		RR				2.0 (1.29–3.08)	0.002			0.194	36.4				0.327				No
Sexual dysfunction	Luhao Liu (2015)		3 cohort studies, 5 case–control studies, 1 cross-sectional study		1272			681		RR				1.87 (1.35–2.58)	< 0.001			0.001	70.1				0.692				No
Osteoporosis	Sikarin Upala (2016)		2 cohort studies, 2 cross-sectional studies		113,922			3141		OR				1.13 (0.60–2.14)	0.703			< 0.001	89.1				0.608^&^				No
Gout	Tingting Shi (2019)		3 cohort studies		154,455			30,109		HR				1.25 (0.91–1.70)	0.162			< 0.001	91				0.876				No
Cancer incidence	Ghanshyam Palamaner Subash Shantha (2015)		5 cohort studies		112,226			904		RR				1.40 (1.01–1.95)	0.04			0.04	60				0.069				Yes
Depression	Cass Edwards (2020)		5 cohort studies		45,056			10,983		RR				2.18 (1.47–2.88)	< 0.001			0.005	72.8				0.667^&^				No
Crash risk	Stephen Tregear (2009)		10 observational studies		10,846			2214		RR				2.43 (1.21–4.89)	0.013			< 0.001	89				0.838^&^				No
Work accidents	Sergio Garbarino (2016)		7 cross-sectional studies		8819			2738		OR				2.18 (1.53–3.10)	< 0.001			0.02	61				0.61				No
Carotid intima-media thickness (CIMT)	Min Zhou (2016)		10 case–control studies, 8 case-sectional studies		1896			1247		SMD				0.88 (0.65, 1.12)	< 0.001			< 0.001	81				0.94				No

^*^p value of significance level

^#^*p* value of *Q* test

^※^*p* value for Egger’s test

^$^The publication bias was assessed using funnel plot

^&^The result was reanalyzed

### Summary effect size

A brief explanation of the effects of the included meta-analysis is given in Table 1. Overall, 111 (82%) of the 136 data reported significant summary outcomes (*p* < 0.05). These associations relate to the outcomes of the following different systems: 29 meta-analyses in cardiovascular disorders, 5 in cerebral and cerebrovascular disease, 4 in mortality, 14 in postoperative complications, 8 in pregnancy-related disorders, 7 in ophthalmic disorders, 11 in digestive disorders, 14 in endocrine and metabolic system, 7 in urological disorders, and 12 in other outcomes. Therefore, it can be concluded that OSA can enhance the risk of disease and have adverse effects on human health.

### Heterogeneity and publication bias

For heterogeneity, 5 results in 5 articles were reanalyzed owing to that they did not exhibit the outcomes of heterogeneity [22, 36, 46, 59, 64]. Among the 136 outcomes including the reanalyzed articles, 47 outcomes showed no heterogeneity between researches (*p* ≥ 0.1 of *Q* test), whereas 69 indicated significant heterogeneity (*p* < 0.1 of *Q* test). However, there were still 20 results in 2 articles that could not be reanalyzed due to the lack of raw data [52, 95], so we could not evaluate their heterogeneity. For publication bias, 76 outcomes demonstrated no statistical evidence on publication bias (*p* ≥ 0.1 of Egger’s test), whereas 25 outcomes presented publication bias (*p* < 0.1 of Egger’s test). There were still 35 results in 9 articles that could not be reanalyzed due to the lack of raw data [45, 52, 54, 55, 87, 92–95], so we could not evaluate their publication bias.

### AMSTAR 2 and summary of evidence

**Fig. 3:**
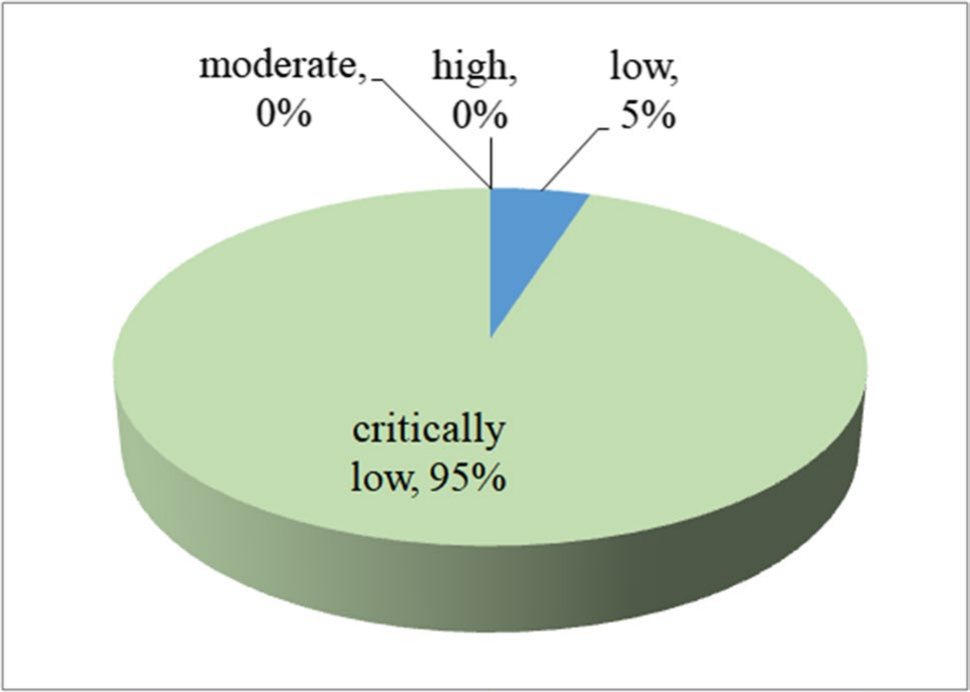
Map of results of AMSTAR 2

The results for the evaluation of the methodological qualities of the 66 included articles are shown in Table 2. Only 3 (5%) studies were determined to be low; the remaining 63 (95%) studies were determined to be critically low (Fig. 3). Based on the AMSTAR 2 criteria, none of the investigations were graded as moderate or high quality.

**Table 2:** Assessments of AMSTAR 2 scores

Reference	AMSTAR 2 checklist																Overall assessment quality
	No. 1	No. 2	No. 3	No. 4	No. 5	No. 6	No. 7	No. 8	No. 9	No. 10	No. 11	No. 12	No. 13	No. 14	No. 15	No. 16	
Xiushi Zhou (2018)	Yes	No	Yes	Partial yes	No	No	Partial yes	Partial yes	Yes	No	Yes	Yes	Yes	Yes	Yes	Yes	Critically low
Xia Wang (2013)	Yes	No	Yes	Partial yes	Yes	Yes	Partial yes	Yes	Yes	No	Yes	Yes	Yes	Yes	Yes	Yes	Critically low
Min Li (2014)	Yes	No	Yes	Partial yes	Yes	Yes	Partial yes	Yes	Yes	No	Yes	No	No	No	No	No	Critically low
Wuxiang Xie (2014)	Yes	No	Yes	Partial yes	Yes	Yes	Partial yes	Yes	Yes	No	Yes	Yes	Yes	Yes	Yes	Yes	Critically low
Chengjuan Xie (2017)	Yes	No	Yes	Partial yes	Yes	Yes	Partial yes	Yes	Yes	No	Yes	No	No	Yes	Yes	Yes	Critically low
Irini Youssef (2018)	Yes	No	No	Partial yes	No	No	Partial yes	No	No	No	Yes	No	No	No	No	No	Critically low
Haifeng Hou (2018)	Yes	Yes	Yes	Partial yes	Yes	Yes	Partial yes	Yes	No	No	Yes	Yes	No	Yes	Yes	Yes	Critically low
Chee Yuan Ng (2011)	Yes	No	Yes	Partial yes	Yes	Yes	Yes	Partial yes	Yes	No	Yes	Yes	Yes	Yes	Yes	No	Critically low
Xiao Wang (2018)	Yes	No	Yes	Partial yes	Yes	Yes	Partial yes	Yes	Yes	No	Yes	Yes	Yes	Yes	Yes	Yes	Critically low
Hua Qu (2018)	Yes	No	Yes	Partial yes	Yes	Yes	Partial yes	Partial yes	Yes	No	Yes	No	Yes	Yes	Yes	Yes	Critically low
Cesare Cuspidi (2020)	Yes	No	No	Partial yes	Yes	Yes	Partial yes	Partial yes	No	No	Yes	No	No	Yes	No	Yes	Critically low
Bo-Lin Ho (2018)	Yes	No	No	Partial yes	Yes	Yes	Yes	Yes	Yes	No	Yes	Yes	Yes	Yes	Yes	Yes	Critically low
Zesheng Wu (2018)	Yes	No	Yes	Partial yes	Yes	Yes	Partial yes	Yes	No	No	Yes	Yes	Yes	Yes	Yes	Yes	Critically low
Yuhong Huang (2019)	Yes	No	No	Partial yes	Yes	Yes	Yes	Yes	Yes	No	Yes	Yes	Yes	Yes	Yes	Yes	Critically low
Anthipa Chokesuwattanaskul (2019)	Yes	No	Yes	Partial yes	No	No	Partial yes	Yes	No	No	Yes	No	No	Yes	Yes	Yes	Critically low
Lei Pan (2016)	Yes	No	Yes	Partial yes	Yes	Yes	Yes	Yes	No	No	Yes	Yes	Yes	Yes	Yes	Yes	Critically low
Xiahui Ge (2013)	Yes	No	Yes	Partial yes	Yes	Yes	Partial yes	Yes	Yes	No	Yes	Yes	Yes	Yes	Yes	Yes	Critically low
Xiaobin Zhang (2017)	Yes	No	Yes	Partial yes	Yes	Yes	Partial yes	Yes	No	No	Yes	No	No	Yes	Yes	Yes	Critically low
Faizi Hai BA (2013)	Yes	No	Yes	Partial yes	Yes	Yes	Partial yes	Partial yes	Yes	No	Yes	Yes	Yes	Yes	Yes	Yes	Critically low
R. Kaw (2012)	Yes	No	Yes	Partial yes	Yes	Yes	Yes	Partial yes	Yes	No	Yes	No	No	Yes	Yes	Yes	Critically low
Ka Ting Ng (2020)	Yes	Yes	Yes	Partial yes	Yes	Yes	Partial yes	Partial yes	Yes	No	Yes	No	No	Yes	Yes	Yes	Critically low
Xinge Zhang (2020)	Yes	No	Yes	Partial yes	Yes	Yes	Partial yes	Partial yes	No	No	Yes	Yes	No	Yes	Yes	Yes	Critically low
Lina Liu (2019)	Yes	No	Yes	Partial yes	Yes	Yes	Partial yes	Partial yes	No	No	Yes	Yes	No	Yes	Yes	Yes	Critically low
Liwen Li (2018)	Yes	No	Yes	Partial yes	Yes	Yes	Partial yes	Yes	No	No	Yes	Yes	No	No	Yes	Yes	Critically low
Ting Xu (2014)	Yes	No	Yes	Partial yes	Yes	Yes	Partial yes	Partial yes	Yes	No	Yes	Yes	Yes	Yes	No	Yes	Critically low
Marco Pellegrini (2020)	Yes	No	Yes	Partial yes	Yes	Yes	Partial yes	Partial yes	Yes	No	Yes	Yes	Yes	Yes	Yes	Yes	Critically low
Xinhua Wu (2015)	Yes	No	Yes	Partial yes	Yes	Yes	Partial yes	Partial yes	No	No	Yes	No	No	Yes	Yes	Yes	Critically low
Leh-Kiong Huon (2016)	Yes	No	Yes	Partial yes	Yes	Yes	Partial yes	Partial yes	No	No	Yes	No	No	No	No	Yes	Critically low
Yong Wu (2015)	Yes	No	Yes	Partial yes	Yes	Yes	Partial yes	Yes	No	No	Yes	Yes	Yes	Yes	Yes	Yes	Critically low
Chris Y.Wu (2018)	Yes	No	Yes	Partial yes	Yes	Yes	Partial yes	Yes	No	No	Yes	No	No	Yes	Yes	Yes	Critically low
Ranran Qie (2020)	Yes	No	Yes	Partial yes	No	No	Partial yes	Yes	No	No	Yes	No	No	Yes	Yes	Yes	Critically low
Xiandong Gu (2018)	Yes	No	Yes	Partial yes	No	No	Partial yes	Yes	No	No	Yes	No	No	Yes	Yes	Yes	Critically low
Wen Bun Leong (2016)	Yes	Yes	Yes	Yes	Yes	Yes	Partial yes	Yes	Yes	No	Yes	Yes	Yes	Yes	Yes	Yes	Low
Zhenliu Zhu (2017)	Yes	No	Yes	Partial yes	Yes	Yes	Partial yes	Partial yes	No	No	Yes	Yes	No	Yes	Yes	Yes	Critically low
Zeng-Hong Wu (2019)	Yes	No	Yes	Partial yes	Yes	Yes	Partial yes	Partial yes	Yes	No	Yes	Yes	Yes	Yes	Yes	Yes	Critically low
Shanshan Jin (2018)	Yes	No	No	Partial yes	Yes	Yes	Partial yes	Yes	No	No	Yes	Yes	No	Yes	Yes	Yes	Critically low
G. Musso (2013)	Yes	No	Yes	Partial yes	Yes	Yes	Partial yes	Partial yes	Yes	No	Yes	No	No	Yes	Yes	Yes	Critically low
Tzong-Yun Ger (2020)	Yes	Yes	Yes	Partial yes	Yes	Yes	Partial yes	Partial yes	Yes	No	Yes	Yes	Yes	No	No	Yes	Critically low
Jiatong Zhou (2019)	Yes	No	Yes	Partial yes	Yes	Yes	Partial yes	Partial yes	No	No	Yes	No	No	Yes	Yes	Yes	Critically low
Yuan Cao (2018)	Yes	No	Yes	Partial yes	Yes	Yes	Partial yes	Yes	No	No	Yes	No	No	No	No	Yes	Critically low
A-Ping Sun (2020)	Yes	No	Yes	Partial yes	Yes	Yes	Partial yes	Partial yes	No	No	Yes	Yes	Yes	Yes	Yes	Yes	Critically low
Luhao Liu (2015)	Yes	No	Yes	Partial yes	Yes	Yes	No	Yes	No	No	Yes	No	No	No	Yes	Yes	Critically low
Sikarin Upala (2016)	Yes	Yes	Yes	Partial yes	Yes	Yes	Partial yes	Yes	Yes	No	Yes	Yes	Yes	Yes	Yes	Yes	Low
Tingting Shi (2019)	Yes	No	Yes	Partial yes	Yes	Yes	Partial yes	Partial yes	No	No	Yes	Yes	No	No	Yes	Yes	Critically low
Tongtong Liu (2020)	Yes	No	Yes	Partial yes	Yes	Yes	Partial yes	Yes	No	No	Yes	No	No	No	Yes	Yes	Critically low
Der-Wei Hwu (2017)	Yes	Yes	Yes	Partial yes	Yes	Yes	Yes	Partial yes	No	No	Yes	No	No	No	No	Yes	Critically low
Ghanshyam Palamaner Subash Shantha (2015)	Yes	No	Yes	Partial yes	Yes	Yes	Partial yes	Partial yes	No	No	Yes	Yes	Yes	Yes	Yes	Yes	Critically low
Shaoyong Xu (2015)	Yes	No	Yes	Partial yes	Yes	Yes	Partial yes	Yes	Yes	No	Yes	Yes	Yes	Yes	Yes	Yes	Critically low
Cass Edwards (2020)	Yes	No	Yes	Partial yes	Yes	Yes	Yes	Partial yes	Yes	No	Yes	Yes	Yes	Yes	Yes	Yes	Critically low
Stephen Tregear (2009)	Yes	No	No	Yes	Yes	Yes	Partial yes	Partial yes	Yes	No	Yes	No	No	Yes	Yes	Yes	Critically low
Sergio Garbarino (2016)	Yes	No	Yes	Partial yes	Yes	Yes	Partial yes	Partial yes	Yes	No	Yes	Yes	Yes	Yes	Yes	Yes	Critically low
Cheng-Lin Sun (2016)	Yes	No	Yes	Partial yes	Yes	Yes	Partial yes	Yes	No	No	Yes	No	Yes	Yes	Yes	Yes	Critically low
Min Zhou (2016)	Yes	No	Yes	Partial yes	Yes	Yes	Partial yes	Partial yes	No	No	Yes	Yes	No	Yes	Yes	Yes	Critically low
Guang Song (2020)	Yes	No	Yes	Partial yes	Yes	Yes	Partial yes	Partial yes	No	No	Yes	Yes	No	Yes	Yes	Yes	Critically low
LeiYu (2019)	Yes	No	No	Partial yes	Yes	Yes	Partial yes	Partial yes	No	No	Yes	Yes	No	Yes	Yes	Yes	Critically low
Abdirashit Maripov (2017)	Yes	No	No	Partial yes	Yes	Yes	Partial yes	Partial yes	No	No	Yes	No	No	Yes	Yes	Yes	Critically low
Rui-Heng Zhang (2020)	Yes	No	No	Partial yes	Yes	Yes	Partial yes	Yes	Yes	No	Yes	No	No	Yes	Yes	Yes	Critically low
De-Lei Kong (2016)	Yes	No	Yes	Partial yes	Yes	Yes	Partial yes	Partial yes	Yes	No	Yes	Yes	No	Yes	Yes	Yes	Critically low
Rashid Nadeem (2014)	Yes	No	Yes	Partial yes	Yes	Yes	Partial yes	No	No	No	Yes	Yes	No	Yes	Yes	Yes	Critically low
Mi Lu (2019)	Yes	No	Yes	Partial yes	Yes	Yes	Partial yes	Partial yes	Yes	No	Yes	Yes	No	Yes	Yes	Yes	Critically low
Reza Fadaei (2020)	Yes	No	Yes	Partial yes	Yes	Yes	Partial yes	Partial yes	Yes	No	Yes	No	No	Yes	Yes	Yes	Critically low
Fang Lu (2019)	Yes	No	Yes	Partial yes	Yes	Yes	Partial yes	Partial yes	Yes	No	Yes	Yes	No	Yes	Yes	Yes	Critically low
Kun Li (2017)	Yes	No	Yes	Partial yes	Yes	Yes	Partial yes	Partial yes	Yes	No	Yes	Yes	Yes	Yes	Yes	Yes	Critically low
Xingyu Wu (2018)	Yes	No	Yes	Partial yes	Yes	Yes	Partial yes	Partial yes	No	No	Yes	No	No	Yes	Yes	Yes	Critically low
Ze-Ning Jin (2016)	Yes	No	No	Partial yes	Yes	Yes	Partial yes	Yes	Yes	No	Yes	Yes	No	Yes	Yes	Yes	Critically low
Xiaoyan Li (2020)	Yes	Yes	Yes	Partial yes	Yes	Yes	Partial yes	Partial yes	Yes	No	Yes	Yes	Yes	Yes	Yes	Yes	Low

**Fig. 4:**
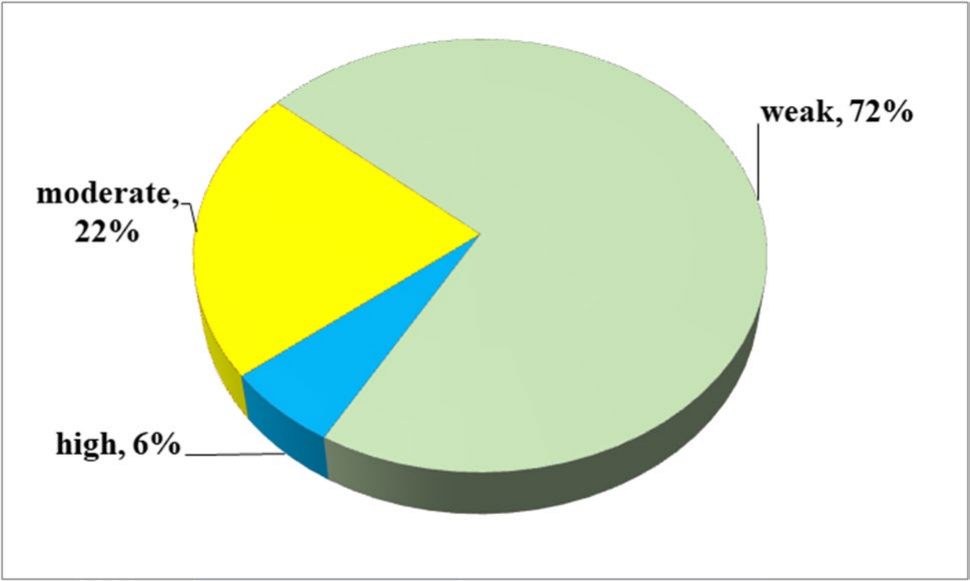
Map of results of evidence assessment

The outcomes of the evidence measurement are shown in Table 3. When a study did not present the result of heterogeneity and publication bias, the corresponding criteria were considered to be not satisfied. Among the 111 statistically significant outcomes, 7 (6%) showed high epidemiologic evidence, 24 (22%) showed moderate epidemiologic evidence, and the remaining 80 (72%) were rated as weak (Fig. 4).

**Table 3:** Detail of results for evidence quality assessing

Outcomes	Reference	Precision of the estimate		Consistency of results	No evidence of small-study effects	Grade
		> 1000 disease cases	*P < *0.001	(*I*^**2** < 50% and Cochran *Q* test *P* > 0.10)^	(*P* > 0.10)	
Cardiovascular disorders						
Aortic dissection	Xiushi Zhou (2018)	Yes	No	Yes	Yes	Weak
Cardiovascular disease (^CVD^)	Xia Wang (2013)	Yes	Yes	Yes	No	Moderate
Stroke	Min Li (2014)	No	Yes	No	Yes	Weak
Ischemic heart disease (IHD)	Wuxiang Xie (2014)	No	No	Yes	No	Weak
Coronary heart disease (CHD)	Chengjuan Xie (2017)	Yes	No	No	Yes	Weak
Major adverse cardiac events (MACEs)	Chengjuan Xie (2017)	Yes	Yes	No	Yes	Moderate
Atrial fibrillation	Irini Youssef (2018)	Yes	Yes	No	No	Weak
Resistant hypertension	Haifeng Hou (2018)	No	Yes	Yes	Yes	Moderate
Essential hypertension	Haifeng Hou (2018)	Yes	Yes	Yes	No	Moderate
Atrial fibrillation recurrence after catheter ablation	Chee Yuan Ng (2011)	No	No	No	Yes	Weak
Major adverse cardiovascular event (MACE) after PCI	Xiao Wang (2018)	Yes	Yes	No	No	Weak
Myocardial infarction(MI) after PCI	Hua Qu (2018)	Yes	No	Yes	Yes	Weak
Coronary revascularization after PCI	Hua Qu (2018)	Yes	Yes	Yes	Yes	High
Left ventricular hypertrophy (LVH)	Cesare Cuspidi (2020)	Yes	Yes	No	No	Weak
Left ventricular diastolic diameter (LVEDD)	LeiYu (2019)	No	Yes	Yes	Yes	Moderate
Left ventricular systolic diameter (LVESD)	LeiYu (2019)	No	No	Yes	Yes	Weak
Left ventricular mass (LVM)	LeiYu (2019)	No	Yes	No	Yes	Weak
Left ventricular ejection fraction (LVEF)	LeiYu (2019)	No	No	No	No	Weak
Left atrial diameter (LAD)	LeiYu (2019)	No	Yes	Yes	No	Weak
Left atrial diameter volume index (LAVI)	LeiYu (2019)	No	Yes	Yes	Yes	Moderate
Right ventricular internal diameter (RVID)	Abdirashit Maripov (2017)	No	Yes	No	No	Weak
Right ventricular free wall thickness (RVWT)	Abdirashit Maripov (2017)	No	Yes	No	Yes	Weak
Right ventricular myocardial performance index (RV MPI)	Abdirashit Maripov (2017)	No	Yes	No	Yes	Weak
Tricuspid annular systolic velocity (RV S′)	Abdirashit Maripov (2017)	No	No	No	Yes	Weak
Tricuspid annular plane systolic excursion (TAPSE)	Abdirashit Maripov (2017)	No	Yes	No	Yes	Weak
Right ventricular fractional area change (RA FAC)	Abdirashit Maripov (2017)	No	No	No	No	Weak
Epicardial adipose tissue (EAT) thickness	Guang Song (2020)	No	Yes	No	Yes	Weak
Coronary flow reserve (CFR)	Rui-Heng Zhang (2020)	No	Yes	No	Yes	Weak
Systolic blood pressure (SBP)	De-Lei Kong (2016)	No	Yes	Yes	NA	Weak
Cerebral and cerebrovascular disease						
Cerebral white matter changes	Bo-Lin Ho (2018)	No	Yes	No	Yes	Weak
Cerebrovascular (CV) disease	Zesheng Wu (2018)	Yes	No	No	No	Weak
White matter hyperintensities (WMH)	Yuhong Huang (2019)	Yes	Yes	No	No	Weak
Silent brain infarction (SBI)	Yuhong Huang (2019)	Yes	No	No	Yes	Weak
Asymptomatic lacunar infarction (ALI)	Anthipa Chokesuwattanaskul (2019)	No	No	Yes	Yes	Weak
Mortality						
All-cause mortality	Lei Pan (2016)	Yes	No	No	No	Weak
Cardiovascular mortality	Xiahui Ge (2013)	No	Yes	Yes	Yes	Moderate
All-cause death after PCI	Xiao Wang (2018)	Yes	No	Yes	Yes	Weak
Cardiac death after PCI	Hua Qu (2018)	Yes	No	Yes	Yes	Weak
Postoperative complications						
Postoperative respiratory failure	Faizi Hai BA (2013)	Yes	Yes	Yes	Yes	High
Postoperative cardiac events	Faizi Hai BA (2013)	Yes	No	Yes	Yes	Weak
Postoperative desaturation	R. Kaw (2012)	Yes	No	No	No	Weak
Postoperative ICU transfer	R. Kaw (2012)	Yes	No	No	No	Weak
Postoperative composite endpoints of postoperative cardiac or cerebrovascular complications	Ka Ting Ng (2020)	Yes	Yes	No	NA	Weak
Postoperative myocardial infarction	Ka Ting Ng (2020)	NA	Yes	Yes	NA	Weak
Postoperative atrial fibrillation	Ka Ting Ng (2020)	NA	Yes	No	NA	Weak
Postoperative composite endpoints of pulmonary complications	Ka Ting Ng (2020)	NA	Yes	No	NA	Weak
Postoperative pneumonia	Ka Ting Ng (2020)	NA	No	No	NA	Weak
Postoperative 30-day mortality	Ka Ting Ng (2020)	NA	No	Yes	NA	Weak
Postoperative acute kidney injury	Ka Ting Ng (2020)	NA	Yes	No	NA	Weak
Postoperative delirium	Ka Ting Ng (2020)	NA	Yes	Yes	NA	Weak
Postoperative venoembolism	Ka Ting Ng (2020)	NA	No	No	NA	Weak
Postoperative length of hospital stay (days)	Ka Ting Ng (2020)	NA	No	No	NA	Weak
Pregnancy-related disorders						
Gestational diabetes mellitus (GDM)	Xinge Zhang (2020)	Yes	No	No	Yes	Weak
C-section	Lina Liu (2019)	NA	Yes	No	NA	Weak
Pregnancy-related wound complication	Lina Liu (2019)	NA	Yes	Yes	NA	Weak
Pregnancy-related pulmonary edema	Lina Liu (2019)	NA	Yes	Yes	NA	Weak
Gestational hypertension	Liwen Li (2018)	Yes	No	Yes	Yes	Weak
Preeclampsia	Liwen Li (2018)	Yes	Yes	No	Yes	Moderate
Preterm birth	Liwen Li (2018)	Yes	No	No	Yes	Weak
Neonatal intensive care unit (NICU) admission	Ting Xu (2014)	No	Yes	No	No	Weak
Ophthalmic disorders						
Diabetic retinopathy (DR)	Zhenliu Zhu (2017)	No	Yes	No	Yes	Weak
Keratoconus	Marco Pellegrini (2020)	Yes	No	Yes	No	Weak
Glaucoma	Xinhua Wu (2015)	Yes	Yes	No	Yes	Moderate
Floppy eyelid syndrome (FES)	Leh-Kiong Huon (2016)	No	Yes	Yes	Yes	Moderate
Nonarteritic anterior ischemic optic neuropathy (NAION)	Yong Wu (2015)	No	No	No	Yes	Weak
Central serous chorioretinopathy (CSCR)	Chris Y.Wu (2018)	Yes	No	Yes	Yes	Weak
Retinal nerve fiber layer (RNFL) thickness	Cheng-Lin Sun (2016)	No	No	No	Yes	Weak
Digestive disorders						
Gastroesophageal reflux disease	Zeng-Hong Wu (2019)	Yes	No	No	No	Weak
Steatosis	Shanshan Jin (2018)	Yes	Yes	Yes	Yes	High
Lobular inflammation	Shanshan Jin (2018)	No	Yes	Yes	Yes	Moderate
Ballooning degeneration	Shanshan Jin (2018)	No	No	Yes	Yes	Weak
NAFLD defined by liver histology	G. Musso (2013)	No	Yes	Yes	Yes	Moderate
NAFLD defined by radiology	G. Musso (2013)	No	Yes	Yes	Yes	Moderate
NAFLD defined by AST elevation	G. Musso (2013)	No	Yes	Yes	Yes	Moderate
NAFLD defined by ALT elevation	G. Musso (2013)	No	Yes	Yes	Yes	Moderate
Nonalcoholic steatohepatitis (NASH)	G. Musso (2013)	No	Yes	Yes	Yes	Moderate
Fibrosis	G. Musso (2013)	No	Yes	Yes	Yes	Moderate
Alanine transaminase (ALT)	Shanshan Jin (2018)	Yes	Yes	Yes	Yes	High
Endocrine and metabolic system disorders						
Type 2 diabetes (T2DM)	Ranran Qie (2020)	Yes	Yes	No	Yes	Moderate
Metabolic syndrome (MS)	Shaoyong Xu (2015)	Yes	Yes	Yes	Yes	High
Fasting blood glucose (FBG)	De-Lei Kong (2016)	Yes	Yes	No	NA	Meak
Total cholesterol (TC)	Rashid Nadeem (2014)	NA	No	NA	NA	Weak
Low-density lipoprotein (LDL)	Rashid Nadeem (2014)	NA	No	NA	NA	Weak
High-density lipoprotein (HDL)	Rashid Nadeem (2014)	NA	Yes	NA	NA	Weak
Triglyceride (TG)	Rashid Nadeem (2014)	NA	Yes	NA	NA	Weak
Adiponectin	Mi Lu (2019)	No	Yes	No	No	Weak
Oxidized low-density lipoprotein (Ox-LDL)	Reza Fadaei (2020)	No	No	No	Yes	Weak
Fibrinogen	Fang Lu (2019)	Yes	Yes	No	Yes	Moderate
Homocysteine	Kun Li (2017)	No	No	No	Yes	Weak
Advanced glycation end products (AGEs)	Xingyu Wu (2018)	No	Yes	No	NA	Weak
Angiotensin II (AngII)	Ze-Ning Jin (2016)	No	Yes	No	Yes	Weak
Serum vitamin D	Xiaoyan Li (2020)	Yes	Yes	No	NA	Weak
Urological disorders						
Diabetic kidney disease (DKD)	Wen Bun Leong (2016)	Yes	No	Yes	Yes	Weak
Microalbuminuria	Tongtong Liu (2020)	No	Yes	Yes	Yes	Moderate
Chronic kidney disease (CKD)	Der-Wei Hwu (2017)	Yes	Yes	No	No	Weak
Serum uric acid level	Tingting Shi (2019)	Yes	Yes	No	No	Weak
Serum cystatin C	Tongtong Liu (2020)	No	Yes	Yes	Yes	Moderate
Estimated glomerular filtration rate (eGFR)	Tongtong Liu (2020)	No	No	No	Yes	Weak
Albumin/creatinine ratio (ACR)	Tongtong Liu (2020)	No	Yes	No	Yes	Weak
Other outcomes						
Diabetic neuropathy	Xiandong Gu (2018)	No	No	No	Yes	Weak
Psoriasis	Tzong-Yun Ger (2020)	Yes	Yes	Yes	Yes	High
Nocturia	Jiatong Zhou (2019)	No	Yes	No	No	Weak
Parkinson’s disease	A-Ping Sun (2020)	Yes	Yes	Yes	Yes	High
Erectile dysfunction	Luhao Liu (2015)	No	No	No	No	Weak
Female sexual dysfunction	Luhao Liu (2015)	No	No	Yes	Yes	Weak
Sexual dysfunction	Luhao Liu (2015)	No	Yes	No	Yes	Weak
Cancer incidence	Ghanshyam Palamaner Subash Shantha (2015)	No	No	No	No	Weak
Depression	Cass Edwards (2020)	Yes	Yes	No	Yes	Moderate
Crash risk	Stephen Tregear (2009)	Yes	No	No	Yes	Weak
Work accidents	Sergio Garbarino (2016)	Yes	Yes	No	Yes	Moderate
Carotid intima-media thickness (CIMT)	Min Zhou (2016)	Yes	Yes	No	Yes	Moderate

## Discussion

In the current umbrella review, we identified 66 meta-analyses of observational studies and evaluated the current evidence supporting an association between OSA and various health outcomes. Also, we provide an extensive overview of the available evidence and critically evaluate the methodological quality of the meta-analyses and the quality of evidence for all the reported associations. OSA increased the risk of 111 health outcomes, including cardiovascular disorders, cerebral and cerebrovascular disease, mortality, postoperative complications, pregnancy-related disorders, ophthalmic disorders, digestive disorders, endocrine and metabolic system disorders, urological disorders, and other outcomes. The evidence quality was graded as high only for coronary revascularization after PCI, postoperative respiratory failure, steatosis, ALT elevation, MS, psoriasis, and Parkinson’s disease. The evidence quality was either moderate or low for the other associations. Furthermore, this umbrella review showed there were no considerable associations between OSA and 25 health outcomes.

Among the 111 outcomes, 54 outcomes had serious heterogeneity between studies. These possible confounding parameters (e.g., sex, body mass index, age, method of assessing OSA, OSA severity, smoking, alcohol drinking, the region of study, and follow-up period) may be the cause of heterogeneity. Substantial heterogeneity led to unreliable results. Of the 111 health outcomes, 23 outcomes possessed a remarkable publication bias, demonstrating that some negative achievements were not presented. Several reasons were leading to publication bias. First, when people start a study, they tend to assume that a positive result may ensure their work complies with the hypothesis during publication. Second, positive results have a higher probability of being published compared to negative results. Third, the study population is only a small fraction of the actual population with the disease. According to AMSTAR 2 criteria, 95% of the studies included in this umbrella analysis had “critically low” methodological quality. The critical flaws considered the absence of a registered protocol, the absence of the risk of bias in the considered investigations, and the absence of consideration of the risk of bias in the included investigations when interpreting or discussing the achieved outcomes of each study. Moreover, none of the meta-analyses in this study explained details of the funding source that had supported the work. The majority of the evaluated meta-analyses had considerable heterogeneity and small-study impacts; these were the main reasons for the evidence rating downgrade.

An umbrella review is a more beneficial method compared to a normal systematic review or meta-analysis due to it representing an overall illustration of achievements for phenomena or special questions [96]. To our knowledge, we are the first to use this method to present a comprehensive critical literature appraisal on published associations between OSA and diverse health information. Also, our two authors systematically searched four scientific databases using a strong search strategy with clearly defined eligibility criteria and data extraction parameters. The quality of included systematic reviews was also evaluated through AMSTAR 2. This is a benchmark methodological quality measurement that is utilized to assessing the quality of the methods utilized for meta-analyses. Furthermore, we graded the epidemiologic evidence conforming to established, prespecified criteria. Its criteria included an assessment of heterogeneity, publication bias, and precision of the estimate, which is more objective than the GRADE system criteria.

There are some limitations in our umbrella review. First, in this analysis, we explained associations evaluated through the meta-analyses of observational investigations. In doing so, we may have missed other health outcomes that have not yet been investigated by meta-analyses. Second, this umbrella analysis included systematic reviews and meta-analyses that were only published in English. The potential missing information in other languages could influence the assessment outcomes. Third, the majority of the meta-analyses had heterogeneity; observational researches are susceptible to uncertainty and confounding bias.

## Conclusions

The associations between OSA and an extensive range of health information have been broadly reported in many meta-analyses. Based on our umbrella review, 66 meta-analyses explored 136 unique outcomes, only 7 outcomes showed a high level of epidemiologic evidence with statistical significance. OSA could be associated with the enhanced risk of coronary revascularization after PCI, postoperative respiratory failure, steatosis, ALT elevation, MS, psoriasis, and Parkinson’s disease. Overall, OSA is harmful to human health but will need further exploration on this topic with high-quality prospective studies.

## Data Availability

The data used to support the findings of this study are included within the article. The primary data used to support the findings of this study are available from the corresponding author upon request.
